# Individualized nutritional care including adherence support improves health-related quality of life in individuals with severe chronic obstructive pulmonary disease: a randomized controlled trial

**DOI:** 10.1186/s12955-026-02482-3

**Published:** 2026-01-30

**Authors:** Maria H. Hegelund, Christian Ritz, Mette F. Olsen, Christian Mølgaard, Thyge L. Nielsen, Andreas V. Jensen, Christian Søborg, Lone Braagaard, Rikke Krogh-Madsen, Birgitte Lindegaard, Daniel Faurholt-Jepsen

**Affiliations:** 1 Department of Pulmonary and Infectious Diseases, Copenhagen University Hospital – North Zealand, Dyrehavevej 29, Hillerød, 3400 Denmark; 2 Department of Infectious Diseases, Copenhagen University Hospital - Rigshospitalet, Copenhagen, Denmark; 3 National Institute of Public Health, University of Southern Denmark, Copenhagen, Denmark; 4 Department of Nutrition, Exercise and Sports, University of Copenhagen, Frederiksberg, Denmark; 5 Department of Infectious Diseases, Copenhagen University Hospital - Hvidovre and Amager, Hvidovre, Denmark; 6 Center for Physical Activity Research, Copenhagen University Hospital - Rigshospitalet, Copenhagen, Denmark; 7 Department of Clinical Medicine, University of Copenhagen, Copenhagen, Denmark

**Keywords:** Chronic obstructive pulmonary disease, Health-related quality of life, Individualized nutritional care, Adherence support, Functional ability

## Abstract

**Background:**

Undernutrition and reduced health-related quality of life (HRQoL) are common in severe chronic obstructive pulmonary disease (COPD) and may exacerbate functional decline. This study evaluated whether individualized nutritional care with adherence support improves HRQoL in individuals with severe COPD at risk of undernutrition. Secondary outcomes included physical function, anthropometry, body composition, and dietary intake.

**Methods:**

In this 3-month, single-center, open-label randomized controlled trial, 87 adults with severe COPD were randomized 1:1 to individualized nutritional care or standard care. The intervention comprised tailored dietary plans, adherence support (phone calls, reminders), optional oral nutritional supplements and weight diary. The primary outcome was HRQoL (EQ-5D-5 L). Secondary outcomes were disease-specific HRQoL (CAT), chair stand test, grip strength, anthropometry, body composition, and protein and energy intake. Analyses followed an intention-to-treat approach using linear mixed-effects models.

**Results:**

Of 674 individuals screened, 91 were enrolled; of whom 87 completed the baseline visit; 44 received the intervention and 43 standard care, with 78 (90%) completing follow-up. At 3 months, the intervention improved HRQoL versus standard care (mean difference in EQ-5D-5 L utility index 0.053; 95% CI 0.003–0.103), driven by better *mobility*, *self-care*, and *usual activities*. Functional gains were supported by more chair stands (mean difference 0.80; 95% CI 0.05–1.55). Protein (+ 16 g/day) and energy intake (+ 326 kcal/day) increased, while no significant differences were observed in disease-specific HRQoL, anthropometry, or body composition. Benefits were greater in undernourished, frail, solitary-living participants, and men.

**Conclusion:**

Individualized nutritional care with adherence support improved HRQoL, particularly physical functioning, in individuals with severe COPD at risk of undernutrition, supporting its consideration in clinical practice.

**Trial registration:**

The study was registered on ClinicalTrials (NCT04873856) in April 2021.

**Supplementary Information:**

The online version contains supplementary material available at 10.1186/s12955-026-02482-3.

## Introduction

Chronic obstructive pulmonary disease (COPD) is a major global health concern. In Denmark, approximately 50,000 individuals have severe COPD [1]. Low health-related quality of life (HRQoL) is common in severe COPD due to high symptom burden, low functional ability, anxiety, and depression [2]. Undernutrition, characterized by an insufficient intake relative to biological needs, is also prevalent in COPD [3]. Undernutrition and COPD may be bidirectionally linked: undernutrition can worsen disease progression [3], while COPD-related low appetite and dyspnoea contribute to inadequate food intake [3]. This combination is often associated with reduced mobility and further reductions in HRQoL [4, 5]. Acute exacerbations and infections exacerbate these problems, often causing unintentional weight loss that is difficult to reverse without nutritional support [6, 7]. Increased energy expenditure due to breathing difficulties further complicates nutritional balance [3, 7]. Anxiety and depression are common but often overlooked in COPD, with negative effects on HRQoL, functional ability, and social interactions [8–12].

Systematic reviews suggest that dietary therapy in COPD can improve food intake, anthropometric measures, functional capacity, and HRQoL [13, 14]. However, most studies are over 30 years old, with small sample sizes (6 out of 8 studies included fewer than 30 participants) [14], and only one RCT included individuals with severe COPD, with just 10 participants [15]. Nutritional care remains underprioritized in COPD treatment [16], and despite evidence of potential benefits, interventions are still insufficiently developed and evaluated.

We hypothesized that individualized nutritional care, including adherence support, would improve HRQoL compared with standard care in individuals with severe COPD. The primary outcome was HRQoL, while secondary outcomes included disease-specific quality of life, anthropometric measures, body composition, functional ability, and energy and protein intake.

## Methods

### Trial design

This single-center, open-label randomized controlled trial (RCT) compared individualized nutritional care, including adherence support, with standard care in adults with severe COPD. Participants were randomly allocated 1:1 to intervention or control. Enrollment was completed by March 2023.

### Participants and recruitment

Potential participants were identified from hospital electronic medical records. Random selection was performed using R [17]. Additional recruitment included oral invitations during hospitalization or outpatient visits at the department, primarily for conditions related to COPD, including acute exacerbations and respiratory tract infections. Eligible participants were ≥ 35 years, had severe COPD, could eat orally, lived at home, were undernourished or at risk of undernutrition, and were clinically stable, defined as having no current acute exacerbation or respiratory tract infection at the time of the enrollment. Participants could be informed about the study during hospitalization, but final enrollment and baseline assessment were postponed until clinically stability was achieved. Exclusion criteria included inability to provide informed consent, active solid cancer, severe renal failure, or severe alcohol abuse.

### Setting

The study was conducted at the Department of Pulmonary and Infectious Diseases, Copenhagen University Hospital – North Zealand, Denmark. Baseline and follow-up data were collected in participants’ homes by the study coordinator.

### Standard care

At the pulmonary outpatient clinic, standard care for severe COPD includes assignment to a specialized nurse, annual physician–nurse consultations covering disease progression, symptom management, prognosis, and advance care planning, telephone access to specialized nurses on weekdays, and periodic rehabilitation programs focused on exercise and COPD education, with limited focus on nutrition [18–20].

### Intervention

**Fig. 1:**
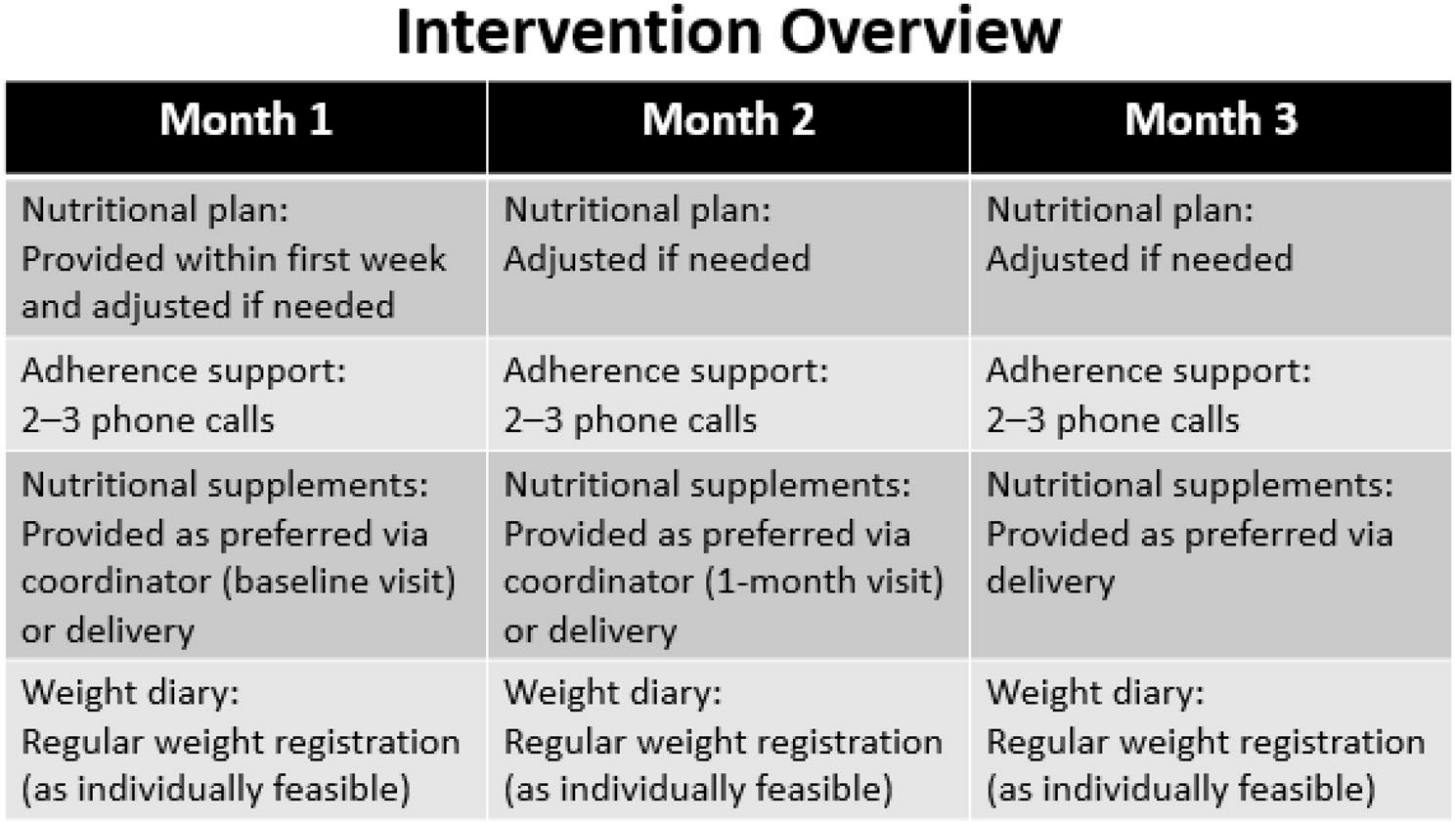
Schematic illustration of the intervention

The 3-month intervention, illustrated in Fig. 1, was designed based on prior studies showing that individualized nutritional care improves intake, functional outcomes, and quality of life in COPD and older adults at risk of undernutrition [7, 21–23]. The intervention was delivered by the study coordinator and included:

- *Individualized nutritional plan*: Nutritional plans were tailored using baseline weight and dietary intake assessed by 24-hour dietary recall. Protein intake was targeted at approximately 1.5 g/kg/day [23], and energy intake at 30–45 kcal/kg/day depending on nutritional status and clinical need [7]. Plans were adapted to adress individual challenges such as dyspnea during meals, low appetite or fatigue.
- *Adherence support*: Evidence-based strategies including phone calls every 7–14 days, a fridge magnet reminder, and encouragement for regular weight tracking, designed to enhance engagement, support self-monitoring, and overcome common barriers to adherence in chronic disease nutritional interventions [7, 24–26]. During adherence calls, nutritional plans were adjusted based on participant progress, weight changes and, dietary intake and reported challenges (e.g., swallowing difficulties, dyspnea).
- *Supplementation*: Participants were offered oral nutritional supplements available through the hospital when needed to support achievement of protein and energy targets. For participants who declined supplements, alternative strategies were provided including guidance on high-protein and energy-dense foods and recipes.

The intervention followed a predefined protocol specifying assessment, target setting, follow-up frequency, and criteria for adjustment, while allowing flexibility to accommodate individual needs (Fig. 1).

After trial conduct, the original non-adherence definition [27] was found difficult to measure reliably. Adherence was therefore defined a priori, prior to data analysis, as ≥ 6 phone contacts, ≥ 4 dietary records, and an average energy and protein intake of ≥ 30 kcal/kg/day and ≥ 1 g/kg/day, with participants not meeting these criteria classified as low adherence.

### Sample size calculation

No prior studies reported EQ-5D-5 L outcomes in a similar population. A prior COPD study using the Chronic Respiratory Disease Questionnaire (CRQ) reported a 10-point improvement in disease-specific HRQoL with a standard deviation 17 [28]. Despite differences between CRQ and EQ-5D-5 L, these data were used as a proxy. A total of 60 participants per group was estimated to provide 80% power at α = 0.05, assuming 20% drop-out.

### Randomization and blinding

Participants were randomized using computer-generated block randomization in R. Allocation concealment was managed by an independent researcher. Group assignment was revealed to the study coordinator and participants after baseline assessment.

### Data collection

#### Nutritional risk

Assessed using NRS-2002 (≥ 3 indicates risk) [29] and MNA-SF (scores 8–11: risk; 0–7: undernutrition) [30]. Participants meeting risk criteria on either tool were eligible if other inclusion criteria were met.

#### Baseline characteristics

Baseline characteristics included demographics, clinical factors, lifestyle, and physical and functional status. Demographics and social factors comprised age, sex, and living situation (solitary vs. cohabiting). Clinical characteristics included COPD diagnosis by spirometry (post-bronchodilator FEV1/FVC < 0.70), disease severity according to GOLD ABCD criteria, with severe COPD defined as GOLD 3–4 or GOLD 2 with high symptom/exacerbation burden [31]. Comorbidity burden was assessed using the Charlson Comorbidity Index, including both total number and specific comorbidities, while home oxygen use and presence of emphysema were obtained from medical records. Lifestyle factors included alcohol intake (categorized as within or above recommendations) and smoking status (never, previous, or current). Physical and functional status was assessed using the FRAIL scale (robust, pre-frail, frail) [32] and undernutrition defined according to ESPEN criteria, considering age, BMI, weight loss, and fat-free mass index (FFMI) [33].

### Outcomes

#### Primary outcome

Generic HRQoL was assessed using the Danish EQ-5D-5 L, covering mobility, self-care, usual activities, pain/discomfort, and anxiety/depression. Each domain was scored 1–5 and converted to a utility index (range − 0.624 to 1.000). Participants also rated overall health on the EQ VAS (0–100) [34].

#### Secondary and other outcomes

Disease-specific HRQoL was assessed with the COPD Assessment Test (CAT; 8 items, score 0–40, higher scores = worse HRQoL) [35, 36]. Anthropometry included height (from medical records, confirmed by participants), weight (0.1 kg, TANITA DC 430 SMA, Denmark), BMI (kg/m²), and waist, hip, and mid-upper arm circumferences (0.1 cm) [37, 38]. Body composition (FFMI, FMI (kg/m²)) was derived from bioelectrical impedance (TANITA DC 430 SMA). Nutritional intake (protein, energy) was estimated from 24-h recalls [39] and calculated using MADlog (Denmark). Functional measures comprised grip strength (kg; SAEHAN DHD-1 dynamometer, South Korea; 3 trials on dominant hand or 3 per hand if uncertainty of dominant hand, highest value recorded) [40] and the 30-s chair stand test (unmodified version) [41]. Physical activity (min/week) was assessed with the physical activity vital signs questionnaire [42] and categorized as < 150 or ≥ 150 min/week according to recommended levels [43]. Outcomes were assessed at baseline, 1 and 3 months.

#### Deviations from pre-registered outcomes

The trial was pre-registered at ClinicalTrials.gov (NCT04873856). During the study, some pre-specified outcomes could not be reported due to feasibility and data quality issues. Data on oxygen therapy and exacerbations were based on self-report and found to be unreliable, and medical record validation was not feasible; therefore, oxygen use was only reported at baseline. Hospital admissions and mortality, originally planned for 3-month follow-up, will instead be reported at 6- and 12-month follow-up. Anxiety and depression (HADS) were not analyzed as permission for the instrument was not obtained before study start. Finally, accelerometer-based physical activity data were not analyzed due to limited time and resources, although future analyses may include these data; only questionnaire-based physical activity data were used for this manuscript.

### Statistical analysis

Analyses were conducted in Stata (StataCorp 2021, Stata Statistical Software: Release 17, College Station, TX: Stata Corp LLC). Continuous variables are presented as means with standard deviation (SD) or median with interquartile range (IQR), whereas categorical variables were reported as counts (%). Intention-to-treat analyses were conducted using linear mixed-effects models. These models included an intervention-by-time interaction as a fixed-effect, while between-participant variation was modeled with participant-specific random intercepts. Relevant covariates (as fixed effects) included variables with observed baseline imbalance. Pre-defined relevant covariates comprised age, sex, solitary-living, FEV1, smoking status, physical activity level, and the baseline value of the outcome. Per-protocol analyses compared fully adherent participants with the standard care group. Subgroup analyses of HRQoL (EQ utility index and the EQ domains) were conducted for undernourished, frail, solitary-living participants, participants with ≥ 1 cardiovascular comorbidity, and by sex on the primary outcome EQ utility index and the EQ domains. Estimated mean differences at 1 month and 3 months were reported together with the corresponding 95% confidence intervals (CI) and p-values. Statistical significance was declared for p-values below 0.05.

### Ethical considerations and approvals

The study was approved by the local ethics committee (H-20060574), registered at ClinicalTrials.gov (identifier: NCT04873856) in April 2021, and conducted in accordance with the Declaration of Helsinki and the CONSORT guidelines [44]. Permission to use the EQ-5D-5 L and CAT instruments was obtained. Written and oral informed consent was obtained from all participants prior to enrolment.

## Results

**Fig. 2:**
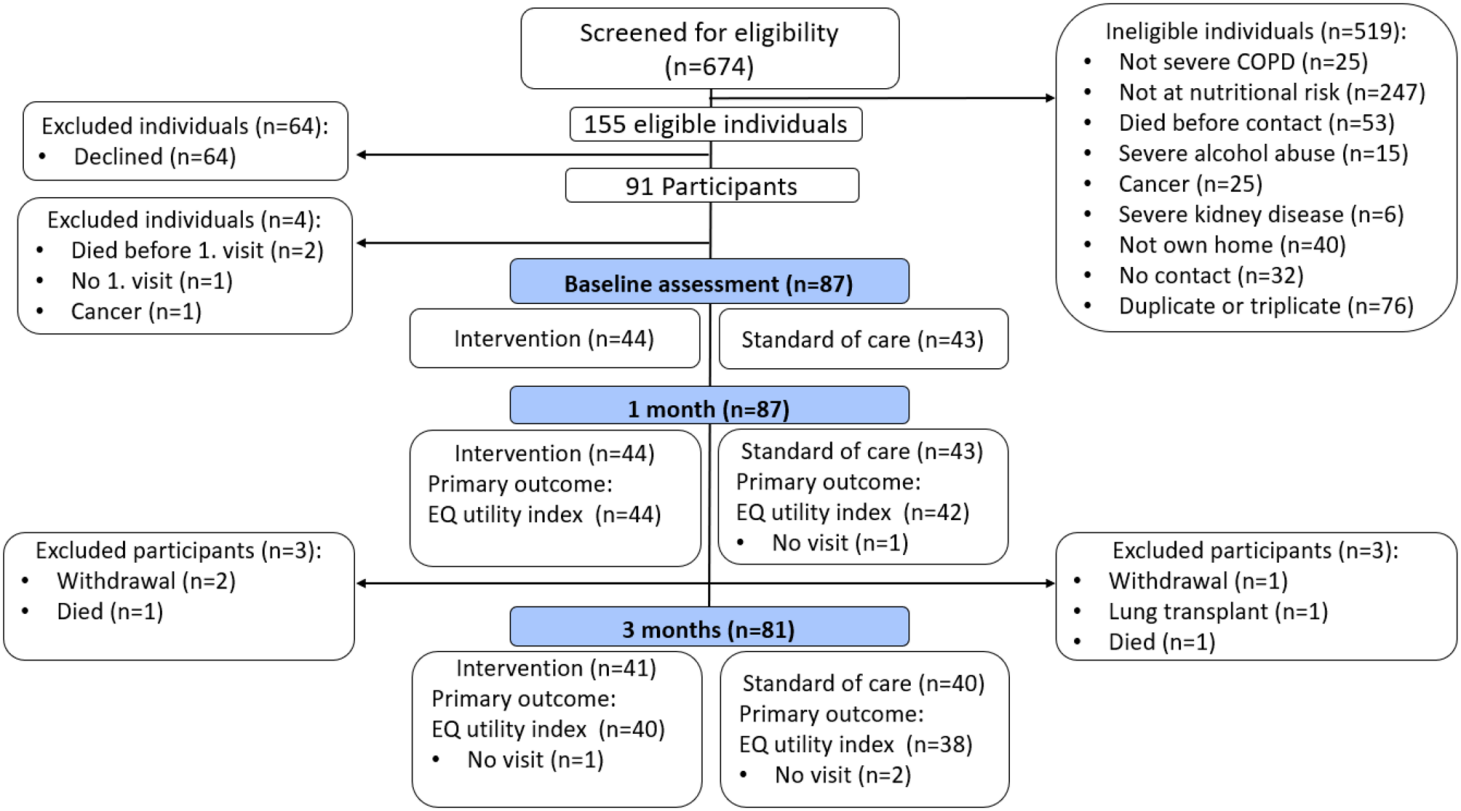
Flow of participants

Of 674 individuals screened, 91 were included between May 2021 and March 2023. Eighty-seven completed the baseline, with 44 randomized to the intervention and 43 to standard care (approximately two-thirds female in each group). Seventy-eight (90%) participants completed the 3-month visit (Fig. 2). At baseline, the median (IQR) FEV1 was 31% (24–42) and 30% used home-based oxygen therapy. Comorbidity was common: 74% had at least one, and 30% had two or more. The most frequent comorbidities were cardiovascular diseases (37%) and osteoporosis (52%). Two thirds of the participants were undernourished and almost 40% were frail (Table 1).

**Table 1:** Baseline characteristics of 87 individuals with severe chronic obstructive pulmonary disease

	Intervention group (*n* = 44)	Control group (*n* = 43)
Age, years	75 ± 7	71 ± 8
Female sex, n (%)	30 (68)	29 (67)
Living alone, n (%)	25 (57)	18 (42)
Alcohol consumption > guidelines, n (%)	9 (20)	12 (27)
**Smoking**,** n (%)**		
Never	3 (7)	1 (2)
Previous	26 (59)	29 (67)
Current	15 (34)	13 (30)
**Lung function**		
Forced expiratory volume in 1 s, %	30.5 (23.5–42)	34 (24–42)
Severe (30–49%), n (%)	20 (45)	19 (44)
Very severe (< 30%), n (%)	24 (55)	24 (56)
Pulmonary emphysema, n (%)	41 (93)	40 (93)
Home-based oxygen therapy, n (%)	13 (30)	13 (30)
**Comorbidity burden**		
Charlson comorbidity index	5 (4–6)	4 (3–5)
Number of comorbidities^#^		
0, n (%)	13 (30)	9 (21)
1, n (%)	15 (34)	23 (53)
≥2, n (%)	15 (36)	11 (26)
**Nutritional risk**		
MNA-SF score ≤ 11, n (%)	43 (98)	42 (98)
NRS2002 score ≥ 3, n (%)	33 (77)	32 (76)
**Physical and functional status**		
Frail, n (%)	21 (48)	12 (29)
Undernourished^##^, n (%)	28 (64)	31 (72)

Data reported as mean ± SD, median (IQR), or n (%). # Number of comorbidities included hypothyroid, Addison Disease, Struma, chronic pancreatitis, sarcoidosis, macroglobulinemia (Waldenstrom), rheumatoid arthritis, dementia, liver disease, cardiovascular disease, osteoporosis, and diabetes. ## Defined according to recommended guidelines by European Society for Clinical Nutrition and Metabolism

### Health-related quality of life (primary outcome)

After three months, the intervention led to a higher HRQoL compared to standard care, with a mean difference (95% CI) in EQ utility index of 0.053 (0.003; 0.103, *p* = 0.04). Improvements were seen in *mobility*,* selfcare*, and *usual activities* with mean differences of -0.543 (-0.901; -0.185, *p* < 0.01), -0.686 (-1.016; -0.356, *p* < 0.01), and − 0.496 (-0.818; -0.175, *p* < 0.01), respectively. No between groups differences was seen in EQ VAS (-4.77, -12-12; 2.59, *p* = 0.20) (Table 2). Subgroup analyses showed benefits in HRQoL among undernourished participants, solitary-living participants, frail participants, and men with mean differences (95% CI) in the EQ utility index of 0.078 (0.013; 0.143, *p* < 0.01), 0.065 (0.005; 0.120, *p* = 0.03), 0.210 (0.137; 0.283, *p* < 0.01), and 0.104 (0.026; 0.181, *p* < 0.01), respectively. Effects across EQ domains varied by subgroup (Table 3). No intervention-related adverse events were observed during the study.

### Other effects after three months

There was no difference between the groups in disease-specific HRQoL (CAT score). After three months, waist circumference was 1.22 cm higher in the intervention group (95% CI 0.01; 2.44, *p* = 0.049). No other anthropometric or body composition effects were observed. Protein and energy intake was higher in the intervention group with mean differences of 16 g (95% CI 6.01; 26.05, *p* < 0.01) and 326 kcal (95% CI 105.08; 547.74, *p* < 0.01), respectively. Functional ability was higher in the intervention group with a higher number of chair stands (mean difference 0.80, 95% CI 0.05; 1.55, *p* = 0.04), while no difference was seen in grip strength (Table 2).

### Effects after one month

No differences were observed after one month in generic or disease-specific HRQoL, anthropometry, body composition, chair stand test, or grip strength. However, protein intake was higher in the intervention group (mean difference 12 g, 95% CI 1.87; 21.29, *p* = 0.02), while energy intake did not differ (Table 2). Subgroup analyses showed early HRQoL benefits: frail participants had higher EQ utility index (0.112, 95% CI 0.039; 0.185, *p* < 0.01) and reported improvements in *mobility*,* self-care*, and *anxiety or depression*. Undernourished participants reported improvements in *usual activities* and *pain or discomfort.* Women reported improvements in *usual activities*. Men reported improvements in *pain or discomfort* (Table 3).

### Adherence and per protocol analyses

Fifteen participants were categorized as low adherent: one died, two had less than six contacts or fewer than four dietary registrations and twelve did not meet the threshold of both protein and energy intake. This corresponds to 83% of participants achieving high adherence to the intervention. Per-protocol analyses showed results largely consistent with the intention-to-treat analyses. Effect sizes (in favor of the intervention) were greater for the EQ utility index, the EQ domains *mobility* and *selfcare*, and energy intake at the 3-month follow-up visit. The mean differences for *usual activities* and protein intake were comparable to those in the intention-to-treat analyses. In contrast, the mean differences for the chair stand test and waist circumference were slightly attenuated and no longer statistically significant (Supplementary Table 1).

**Table 2:** Effect of individualized nutritional intervention including adherence support among individuals with severe chronic obstructive pulmonary disease at risk of undernutrition

Outcome	1 month (*n* = 87)		3 months (*n* = 81)	
	Mean difference (95% CI)	*P*	Mean difference (95% CI)	*P*
**Primary**				
EQ utility index	0.018 (-0.031; 0.066)	0.48	0.053 (0.003; 0.103)	0.04
* EQ mobility*	-0.240 (-0.588; 0.107)	0.18	-0.543 (-0.901; -0.185)	< 0.01
* EQ self-care*	-0.316 (-0.637; 0.005)	0.054	-0.686 (-1.016: -0.356)	< 0.01
* EQ usual activities*	-0.227 (-0.539; 0.085)	0.15	-0.496 (-0.818; -0.175)	< 0.01
* EQ pain or discomfort*	-0.329 (-0.703; 0.046)	0.09	-0.245 (-0.633; 0.143)	0.22
* EQ anxiety or depression*	-0.138 (-0.443; 0.168)	0.38	-0.186 (-0.498; 0.126)	0.24
EQ VAS	-0.86 (-8.00; 6.27)	0.81	-4.77 (-12.12; 2.59)	0.20
**Secondary/other**				
COPD Assessment Test	-0.36 (-1.92; 1.21)	0.66	-0.36 (-1.97; 1.25)	0.66
Body mass index, kg/m^2^	0.09 (-0.17; 0.34)	0.50	0.10 (-0.16; 0.36)	0.44
Fat-free mass index, kg/m^2^	0.05 (-0.12; 0.22)	0.58	-0.01 (-0.20; 0.17)	0.89
Fat mass index, kg/m^2^	0.01 (-0.17; 0.20)	0.89	-0.05 (-0.24; 0.14)	0.59
Grip strength, kg	0.13 (-0.88; 1.15)	0.80	0.09 (-0.94; 1.12)	0.86
Chair Stand Test	0.22 (-0.50; 0.95)	0.55	0.80 (0.05; 1.55)	0.04
Waist circumference, cm	0.80 (-0.38; 1.99)	0.18	1.22 (0.01; 2.44)	0.049
Hip circumference, cm	0.09 (-0.88; 1.06)	0.86	0.68 (-0.32; 1.68)	0.18
Upper-arm circumference, cm	0.04 (-0.41; 0.49)	0.87	0.31 (-0.15; 0.77)	0.18
Protein intake, g	11.58 (1.87; 21.29)	0.02	16.03 (6.01; 26.05)	< 0.01
Energy intake, kcal	155.97 (-59.72; 371.66)	0.16	326.41 (105.08; 547.74)	< 0.01
Physical activity, min/week	26 (-76; 137)	0.18	43 (-149; 64)	0.43

Higher EQ utility index and VAS indicate better quality of life. A positive mean difference reflects a beneficial effect for utility and VAS, while a negative mean difference reflects benefit for domain scores (scored 1–5). The model was adjusted for baseline imbalances (age, grip strength, protein intake, Charlson index), predefined covariates (sex, living alone, FEV1, smoking, physical activity), and the baseline outcome value

**Table 3:** Effect of individualized nutritional intervention including adherence support on health-related quality of life among subgroups of severe chronic obstructive pulmonary disease

Outcome	1 month		3 months	
	Mean difference (95% CI)	*P*	Mean difference (95% CI)	*P*
**Undernourished (** ***n*** ** = 59)**				
EQ utility index	0.031 (0.031–0.093)	0.31	0.078 (0.013; 0.143)	0.02
* EQ mobility*	-0.391 (-0.841; 0.058)	0.09	-0.587 (-1.053; -0.120)	0.01
* EQ self-care*	-0.225 (-0.613;0.163)	0.26	-0.796 (-1.201; -0.391)	< 0.01
* EQ usual activities*	-0.535 (-0.905; -0.166)	< 0.01	-0.886 (-1.271; -0.500)	< 0.01
* EQ pain or discomfort*	-0.523 (-1.001; -0.045)	0.03	-0.302 (-0.803; 0.199)	0.24
* EQ anxiety or depression*	-0.094 (-0.480; 0.293)	0.63	-0.226 (-0.625; 0.173)	0.27
**Solitary-living (** ***n*** ** = 43)**				
EQ utility value	0.049 (-0.008; 0.104)	0.09	0.065 (0.005; 0.124)	0.03
* EQ mobility*	-0.431 (-0.939; 0.078)	0.10	-0.868 (-1.405; -0.331)	< 0.01
* EQ self-care*	-0.282 (-0.660; 0.100)	0.14	-0.796 (-1.200; -0.395)	< 0.01
* EQ usual activities*	-0.198 (-0.694; 0.299)	0.44	-0.539 (-1.061; -0.018)	0.04
* EQ pain or discomfort*	-0.144 (-0.625; 0.336)	0.56	-0.191 (-0.697; 0.315)	0.46
* EQ anxiety or depression*	-0.132 (-0.564; 0.299)	0.55	0.039 (-0.407; 0.484)	0.87
**Cardiovascular comorbidity (** ***n*** ** = 32)**				
EQ utility value	0.021 (-0.054; 0.096)	0.58	0.069 (-0.009; 0.147)	0.08
* EQ mobility*	-0.405 (-1.103; 0.393)	0.26	-0.137 (-0.859; 0.585)	0.71
* EQ self-care*	-0.268 (-0.800; 0.264)	0.32	-0.821 (-1.377; -0.265)	< 0.01
* EQ usual activities*	-0.224 (-0.702; 0.253)	0.36	-0.887 (-1.400; -0.394)	< 0.01
* EQ pain or discomfort*	-0.328 (-0.856; 0.201)	0.23	-0.364 (-0.921; 0.193)	0.20
* EQ anxiety or depression*	-0.014 (-0.430; 0.403)	0.95	-0.017 (-0.415; 0.449)	0.94
**Frail (** ***n*** ** = 33)**				
EQ utility value	0.112 (0.039; 0.185)	< 0.01	0.210 (0.137 0.283)	< 0.01
* EQ mobility*	-0.760 (-1.240; -0.281)	< 0.01	-1.183 (-1.661; -0.704)	< 0.01
* EQ self-care*	-0.706 (-1.211; 0.202)	< 0.01	-1.394 (-1.897; -0.890)	< 0.01
* EQ usual activities*	-0.383 (-0.838; 0.072)	0.10	-1.034 (-1.488; -0.580)	< 0.01
* EQ pain or discomfort*	-0.547 (-1.161; 0.067)	0.08	-0.453 (-1.067; 0.162)	0.15
* EQ anxiety or depression*	-0.717 (-1.152; -0.282)	< 0.01	-0.821 (-1.255; -0.387)	< 0.01
**Women (** ***n*** ** = 59)**				
EQ utility value	0.012 (-0.044; 0.068)	0.68	0.024 (-0.034; 0.082)	0.42
* EQ mobility*	-0.107 (-0.512; 0.298)	0.60	-0.458 (-0.880; -0.0365)	0.03
* EQ self-care*	-0.371 (-0.711; 0.031)	0.03	-0.761 (-1.116; -0.407)	< 0.01
* EQ usual activities*	-0.189 (-0.583; 0.206)	0.35	-0.276 (-0.686; 0.135)	0.19
* EQ pain or discomfort*	-0.219 (-0.662; 0.224)	0.33	-0.008 (-0.456; 0.472)	0.97
* EQ anxiety or depression*	-0.009 (-0.358; 0.340)	0.96	-0.012 (-0.369; 0.345)	0.95
**Men (** ***n*** ** = 28)**				
EQ utility value	0.038 (-0.037; 0.113)	0.32	0.104 (0.026; 0.181)	< 0.01
* EQ mobility*	-0.486 (-1.074; 0.103)	0.11	-0.637 (-1.242; 0.033)	0.04
* EQ self-care*	-0.375 (-1.026; 0.277)	0.26	-0.690 (-1.356; 0.023)	0.04
* EQ usual activities*	-0.271 (-0.705; 0.162)	0.22	-0.892 (-1.337; -0.446)	< 0.01
* EQ pain or discomfort*	-0.598 (-1.115; -0.081)	0.02	-0.697 (-1.229; -0.164)	0.01
* EQ anxiety or depression*	-0.400 (-0.943; 0.143)	0.15	-0.490 (-1.047; 0.067)	0.09

Higher EQ utility index and VAS indicate better quality of life. A positive mean difference reflects a beneficial effect for utility and VAS, while a negative mean difference reflects benefit for domain scores (scored 1–5). The model was adjusted for baseline imbalances (age, grip strength, protein intake, Charlson index), predefined covariates (sex, living alone, FEV1, smoking, physical activity), and the baseline outcome value

## Discussion

This study demonstrates that individually tailored nutritional care including adherence support can improve HRQoL compared to standard care, particularly the physical domains, among individuals with severe COPD at risk of undernutrition. To our knowledge, this is the largest RCT of individualized nutritional care in this patient group.

Consistent with prior work, participants had markedly reduced HRQoL at baseline. The median EQ utility index of 0.65 was lower than in the general older population (0.80) [45] and comparable to previous COPD cohorts [46]. The observed mean difference of 0.053 is clinically meaningful, exceeding published thresholds for minimal important differences in COPD [46, 47]. Importantly, improvements were evident across all physical HRQoL domains and supported by gains in functional ability, as shown by the chair stand test. Functional limitations strongly affect HRQoL and can trigger a downward spiral of fatigue, dependence, social isolation, and psychological distress [48–50]. Our findings suggest that nutritional care may interrupt this trajectory.

Some participants in both groups attended outpatient pulmonary rehabilitation, which typically enhances physical performance. However, nutritional needs are often insufficiently addressed within rehabilitation programs [51]. By deliberately separating the interventions, we ensured that vulnerable individuals unwilling or unable to attend rehabilitation were not excluded. Nevertheless, future studies should explore combined approaches, as integrating tailored nutrition with structured physical training may yield greater improvements. Subgroup analyses indicated that undernourished, frail, and male participants benefitted the most. Although these analyses should be interpreted with caution due to small sample sizes, they underscore vulnerable subgroups that may gain the greatest benefit from individualized care.

The intervention improved nutritional intake but not anthropometry or body composition. This likely reflects baseline heterogeneity: although all participants were undernourished or at risk, BMI ranged from 12.9 to 24.6 kg/m², resulting in divergent nutritional goals. For some, the aim was maintenance; for others, weight gain. In this context, functional improvements may be more meaningful than anthropometric change.

Strengths of this study include the high retention rate, likely facilitated by home-based visits, and the use of EQ-5D-5 L, a validated HRQoL instrument in both Danish and COPD populations [46, 52]. Limitations include the open-label design, as neither participants nor outcome assessors were blinded, which may introduce measurement or reporting bias and increase awareness of dietary behaviors among participants in the control group. However, standardized assessment procedures and the use of validated outcome measures were applied to mitigate this risk. The single-center setting may restrict generalizability. COPD is a heterogeneous condition [53]; responses to nutritional care may therefore vary across disease phenotypes. The intervention was delivered as a multicomponent package, and the study was not powered to evaluate the relative contribution or effectiveness of individual components.

Despite these limitations, our findings underline the need for individualized nutritional care in COPD. Even among those with lower adherence, many participants reported increased awareness of nutritional needs. Current outpatient care often overlooks nutrition due to lack of expertise and clear responsibility [16]. The structured and protocol-based nature of the intervention suggests that similar nutritional care programs could be delivered in routine clinical practice as part of outpatient care by trained healthcare professionals following appropriate training. Given the preventive potential across HRQoL domains, a multidisciplinary approach—combining nutritional care, adherence support, physical training, psychiatry, and pharmacology—may yield broader and more sustained benefits.

## Conclusion

Individualized nutritional care with adherence support improved HRQoL, particularly physical functioning, in individuals with severe COPD at risk of undernutrition. As HRQoL is a central treatment goal in advanced COPD, integration of structured nutritional care into clinical practice should be considered. Given the practical challenges of conducting large-scale randomized trials of individualized nutritional interventions in this population, implementation-focused studies in real-world clinical settings may be particularly relevant.

## Supplementary Information

Below is the link to the electronic supplementary material.

Supplementary Material 1

## Data Availability

The datasets used and/or analysed during the current study are available from the corresponding author on reasonable request.

## Abbreviations

BMI: Body mass index
CI: Confidence intervals
COPD: Chronic obstructive pulmonary disease
EQ: EuroQoL
FEV1: Forced expiratory volume in 1 s
FFMI: Fat-free mass index
FMI: Fat mass index
FVC: Forced vital capacity
HRQoL: Health-related quality of life
IQR: Interquartile range
RCT: Randomized controlled trial

## References

[1] Novo Nordisk Foundation. Major research project will help 50,000 Danes who have chronic obstructive pulmonary disease [Internet]. 2020 [cited 2021 21 February]. Available from: https://novonordiskfonden.dk/en/news/major-research-project-will-help-50000-danes-who-have-chronic-obstructive-pulmonary-disease/.

[2] Gardiner C, Gott M, Payne S, Small N, Barnes S, Halpin D, et al. Exploring the care needs of patients with advanced COPD: an overview of the literature. Respir Med. 2010;104(2):159–65. 19818590 10.1016/j.rmed.2009.09.007

[3] Akner G, Larsson K. Undernutrition state in patients with chronic obstructive pulmonary disease. A critical appraisal on diagnostics and treatment. Respir Med. 2016;117:81–91. 27492517 10.1016/j.rmed.2016.05.023

[4] Katsura H, Yamada K, Kida K. Both generic and disease specific health-related quality of life are deteriorated in patients with underweight COPD. Respir Med. 2005;99(5):624–30. 15823461 10.1016/j.rmed.2004.09.017

[5] Luo Y, Zhou L, Li Y, Guo S, Li X, Zheng J, et al. Fat-Free mass index for evaluating the nutritional status and disease severity in COPD. Respir Care. 2016;61(5):680–8. 26814217 10.4187/respcare.04358

[6] Vermeeren MA, Schols AM, Wouters EF. Effects of an acute exacerbation on nutritional and metabolic profile of patients with COPD. Eur Respir J. 1997;10(10):2264–9. 9387951 10.1183/09031936.97.10102264

[7] Collins PF, Yang IA, Chang YC, Vaughan A. Nutritional support in chronic obstructive pulmonary disease (COPD): an evidence update. J Thorac Dis. 2019;11(Suppl 17):S2230–7. 31737350 10.21037/jtd.2019.10.41 PMC6831917

[8] Hill K, Geist R, Goldstein RS, Lacasse Y. Anxiety and depression in end-stage COPD. Eur Respir J. 2008;31(3):667–77. 18310400 10.1183/09031936.00125707

[9] van Manen JG, Bindels PJ, Dekker FW, CJ IJ, van der Zee JS, Schadé E. Risk of depression in patients with chronic obstructive pulmonary disease and its determinants. Thorax. 2002;57(5):412–6. 11978917 10.1136/thorax.57.5.412 PMC1746339

[10] Mikkelsen RL, Middelboe T, Pisinger C, Stage KB. Anxiety and depression in patients with chronic obstructive pulmonary disease (COPD). A review. Nord J Psychiatry. 2004;58(1):65–70. 14985157 10.1080/08039480310000824

[11] Dalal AA, Shah M, Lunacsek O, Hanania NA. Clinical and economic burden of depression/anxiety in chronic obstructive pulmonary disease patients within a managed care population. Copd. 2011;8(4):293–9. 21827298 10.3109/15412555.2011.586659

[12] Doyle T, Palmer S, Johnson J, Babyak MA, Smith P, Mabe S, et al. Association of anxiety and depression with pulmonary-specific symptoms in chronic obstructive pulmonary disease. Int J Psychiatry Med. 2013;45(2):189–202. 23977821 10.2190/PM.45.2.g PMC4005783

[13] Collins PF, Stratton RJ, Elia M. Nutritional support in chronic obstructive pulmonary disease: a systematic review and meta-analysis. Am J Clin Nutr. 2012;95(6):1385–95. 22513295 10.3945/ajcn.111.023499

[14] Collins PF, Elia M, Stratton RJ. Nutritional support and functional capacity in chronic obstructive pulmonary disease: a systematic review and meta-analysis. Respirology. 2013;18(4):616–29. 23432923 10.1111/resp.12070

[15] Møgelberg N, Tobberup R, Møller G, Godtfredsen NS, Nørgaard A, Andersen JR. High-protein diet during pulmonary rehabilitation in patients with chronic obstructive pulmonary disease. Dan Med J. 2022;69(11). 36331152

[16] Sørensen D, Wieghorst AR, Elbek JA, Mousing CA. Mealtime challenges in patients with chronic obstructive pulmonary disease: who is responsible? J Clin Nurs. 2020;29(23–24):4583–93. 32920956 10.1111/jocn.15491

[17] R Core Team. R: A language and environment for statistical computing. 2020.

[18] Bove DG, Lavesen M, Lindegaard B. Characteristics and health related quality of life in a population with advanced chronic obstructive pulmonary disease, a cross-sectional study. BMC Palliat Care. 2020;19(1):84. 32552723 10.1186/s12904-020-00593-2 PMC7301437

[19] Bove DG, Jellington MO, Lavesen M, Marså K, Herling SF. Assigned nurses and a professional relationship: a qualitative study of COPD patients’ perspective on a new palliative outpatient structure named CAPTAIN. BMC Palliat Care. 2019;18(1):24. 30825878 10.1186/s12904-019-0410-0 PMC6397743

[20] Lavesen M, Marsa KB, Bove DG. A new way of organising palliative care for patients with severe chronic obstructive pulmonary disease. Int J Palliat Nurs. 2018;24(2):64–8. 29469649 10.12968/ijpn.2018.24.2.64

[21] Watterson C, Fraser A, Banks M, Isenring E, Miller M, C S, et al. Evidence based practice guidelines for the nutritional management of malnutrition in adult patients across the continuum of care. Nutr Dietetics. 2009;66:S1-S34.

[22] Baldwin C, Weekes CE. Dietary advice with or without oral nutritional supplements for disease-related malnutrition in adults. Cochrane Database Syst Rev. 2011;2011(9):Cd002008. 21901680 10.1002/14651858.CD002008.pub4 PMC6465043

[23] Deutz NE, Bauer JM, Barazzoni R, Biolo G, Boirie Y, Bosy-Westphal A, et al. Protein intake and exercise for optimal muscle function with aging: recommendations from the ESPEN expert group. Clin Nutr. 2014;33(6):929–36. 24814383 10.1016/j.clnu.2014.04.007 PMC4208946

[24] Fenerty SD, West C, Davis SA, Kaplan SG, Feldman SR. The effect of reminder systems on patients’ adherence to treatment. Patient Prefer Adherence. 2012;6:127–35. 22379363 10.2147/PPA.S26314 PMC3287416

[25] O’Leary K, Liu L, McClure JB, Ralston J, Pratt W. Persuasive reminders for health self-management. AMIA Annu Symp Proc. 2016;2016:994–1003. PMC5333289 28269896

[26] Balasubramanian D, Yoong J, Vrijhoef HJ. Telephone support and adherence in patients with chronic disease–a qualitative review of reviews. Smart Homecare Technol Telehealth. 2015:107 – 18.

[27] Hegelund MH, Ritz C, Nielsen TL, Olsen MF, Søborg C, Braagaard L, et al. Multidimensional individualized nutritional therapy for individuals with severe chronic obstructive pulmonary disease: study protocol for a registry-based randomized controlled trial. Trials. 2023;24(1):86. 36747276 10.1186/s13063-023-07099-1 PMC9900973

[28] Sugawara K, Takahashi H, Kashiwagura T, Yamada K, Yanagida S, Homma M, et al. Effect of anti-inflammatory supplementation with Whey peptide and exercise therapy in patients with COPD. Respir Med. 2012;106(11):1526–34. 22857881 10.1016/j.rmed.2012.07.001

[29] Kondrup J, Allison SP, Elia M, Vellas B, Plauth M. ESPEN guidelines for nutrition screening 2002. Clin Nutr. 2003;22(4):415–21. 12880610 10.1016/s0261-5614(03)00098-0

[30] Rubenstein LZ, Harker JO, Salvà A, Guigoz Y, Vellas B. Screening for undernutrition in geriatric practice: developing the short-form mini-nutritional assessment (MNA-SF). J Gerontol Biol Sci Med Sci. 2001;56(6):M366–72. 10.1093/gerona/56.6.m366 11382797

[31] Global initiative for chronic obstructive lung disease. Pocket Guide to COPD diagnosis, management and prevention: a guide for health care professionals. 2020.

[32] Woo J, Leung J, Morley JE. Comparison of frailty indicators based on clinical phenotype and the multiple deficit approach in predicting mortality and physical limitation. J Am Geriatr Soc. 2012;60(8):1478–86. 22861118 10.1111/j.1532-5415.2012.04074.x

[33] Cederholm T, Bosaeus I, Barazzoni R, Bauer J, Van Gossum A, Klek S, et al. Diagnostic criteria for malnutrition - An ESPEN consensus statement. Clin Nutr. 2015;34(3):335–40. 25799486 10.1016/j.clnu.2015.03.001

[34] Herdman M, Gudex C, Lloyd A, Janssen M, Kind P, Parkin D, et al. Development and preliminary testing of the new five-level version of EQ-5D (EQ-5D-5L). Qual Life Res. 2011;20(10):1727–36. 21479777 10.1007/s11136-011-9903-x PMC3220807

[35] Jones PW, Harding G, Berry P, Wiklund I, Chen WH, Kline Leidy N. Development and first validation of the COPD assessment test. Eur Respir J. 2009;34(3):648–54. 19720809 10.1183/09031936.00102509

[36] Jones PW, Harding G, Wiklund I, Berry P, Tabberer M, Yu R, et al. Tests of the responsiveness of the COPD assessment test following acute exacerbation and pulmonary rehabilitation. Chest. 2012;142(1):134–40. 22281796 10.1378/chest.11-0309

[37] World Health Organization. Waist circumference and Waist–Hip ratio: report of a WHO expert consultation. Geneva, Switzerland: World Health Organization; 2008.

[38] Eaton-Evans J. Nutritional assessment: anthropometry. In: Encyclopedia of Human Nutrition. 3rd ed. Waltham (MA): Academic Press; 2013.

[39] Salvador Castell G, Serra-Majem L, Ribas-Barba L. What and how much do we eat? 24-hour dietary recall method. Nutr Hosp. 2015;31(Suppl 3):46–8. 25719770 10.3305/nh.2015.31.sup3.8750

[40] Vargas-Pinilla OC, Rodríguez-Grande EI. Reproducibility and agreement between three positions for handgrip assessment. Sci Rep. 2021;11(1):12906. 34145312 10.1038/s41598-021-92296-8 PMC8213844

[41] Jones CJ, Rikli RE, Beam WC. A 30-s chair-stand test as a measure of lower body strength in community-residing older adults. Res Q Exerc Sport. 1999;70(2):113–9. 10380242 10.1080/02701367.1999.10608028

[42] Greenwood JL, Joy EA, Stanford JB. The physical activity vital sign: a primary care tool to guide counseling for obesity. J Phys Act Health. 2010;7(5):571–6. 20864751 10.1123/jpah.7.5.571

[43] World Health Organization. WHO guidelines on physical activity and sedentary behaviour: at a glance. Switzerland: Geneva; 2021.

[44] Schulz KF, Altman DG, Moher D. CONSORT 2010 statement: updated guidelines for reporting parallel group randomised trials. BMJ. 2010;340:c332. 20332509 10.1136/bmj.c332 PMC2844940

[45] Grochtdreis T, Dams J, König HH, Konnopka A. Health-related quality of life measured with the EQ-5D-5L: Estimation of normative index values based on a representative German population sample and value set. Eur J Health Econ. 2019;20(6):933–44. 31030292 10.1007/s10198-019-01054-1

[46] Nolan CM, Longworth L, Lord J, Canavan JL, Jones SE, Kon SS, et al. The EQ-5D-5L health status questionnaire in COPD: validity, responsiveness and minimum important difference. Thorax. 2016;71(6):493–500. 27030578 10.1136/thoraxjnl-2015-207782 PMC4893131

[47] Bae E, Choi SE, Lee H, Shin G, Kang D. Validity of EQ-5D utility index and minimal clinically important difference Estimation among patients with chronic obstructive pulmonary disease. BMC Pulm Med. 2020;20(1):73. 32293387 10.1186/s12890-020-1116-z PMC7092534

[48] Kouijzer M, Brusse-Keizer M, Bode C. COPD-related fatigue: impact on daily life and treatment opportunities from the patient’s perspective. Respir Med. 2018;141:47–51. 30053971 10.1016/j.rmed.2018.06.011

[49] Gonçalves B, Lusher J, Cund A, Sime C, Harkess-Murphy E. Understanding the psychosocial burden of living with advanced COPD in context of palliative care: a mixed methods study. J Health Psychol. 2025:13591053251316504. 10.1177/13591053251316504 PMC12534879 40079249

[50] Engström CP, Persson LO, Larsson S, Sullivan M. Health-related quality of life in COPD: why both disease-specific and generic measures should be used. Eur Respir J. 2001;18(1):69–76. 10.1183/09031936.01.00044901 11510808

[51] Holst M, Beck AM, Rasmussen HH, Lange P. Insufficient intake of energy and protein is related to physical functional capacity among COPD patients referred to municipality based pulmonary rehabilitation. Clin Nutr ESPEN. 2019;30:35–41. 30904227 10.1016/j.clnesp.2019.02.009

[52] Jensen MB, Jensen CE, Gudex C, Pedersen KM, Sørensen SS, Ehlers LH. Danish population health measured by the EQ-5D-5L. Scand J Public Health. 2023;51(2):241–9. 34847818 10.1177/14034948211058060 PMC9969307

[53] Lange P, Ahmed E, Lahmar ZM, Martinez FJ, Bourdin A. Natural history and mechanisms of COPD. Respirology. 2021;26(4):298–321. 33506971 10.1111/resp.14007

