# Supplementary material for: Individualized nutritional care including adherence support improves health-related quality of life in individuals with severe chronic obstructive pulmonary disease: a randomized controlled trial
Source: Health Qual Life Outcomes. 2026 Jan 30;24:23. doi: 10.1186/s12955-026-02482-3 (PMC12930726; doi:10.1186/s12955-026-02482-3)
Supplement: Supplementary file 1 — Supplementary Material 1 [file 12955_2026_2482_MOESM1_ESM.docx]

**Supplementary table 1.** Per protocol analysis of the effects of individualized nutritional intervention including adherence support among individuals with severe chronic obstructive pulmonary disease.

|  | **1 month** | | **3 months** | |
| --- | --- | --- | --- | --- |
| Outcome | Mean difference (95% CI) | P | Mean difference (95% CI) | P |
| **Primary** |  |  |  |  |
| EQ utility index | 0.026 (-0.025; 0.078) | 0.32 | 0.068 (0.015; 0.122) | 0.01 |
| *EQ mobility* | -0.31 (-0.68; 0.06) | 0.10 | -0.61 (-0.99; -0.23) | <0.01 |
| *EQ self-care* | -0.32 (-0.66; 0.02) | 0.06 | -0.77 (-1.12; -0.42) | <0.01 |
| *EQ usual activities* | -0.18 (-0.50; 0.15) | 0.29 | -0.51 (-0.84; -0.18) | <0.01 |
| *EQ pain or discomfort* | -0.41 (-0.82; 0.01) | 0.053 | -0.23 (0.636; 0.19) | 0.28 |
| *EQ anxiety or depression* | -0.11 (-0.45; 0.22) | 0.51 | -0.20 (-0.54; 0.14) | 0.26 |
| EQ VAS | -2.18 (-9.92; 5.55) | 0.58 | -4.17 (-12.12; 3.78) | 0.30 |
| **Secondary/other** |  |  |  |  |
| COPD Assessment Test | 0.03 (-1.59; 1.64) | 0.97 | -0.02 (-1.67; 1.64) | 0.99 |
| Body mass index, kg/m^2^ | 0.17 (-0.08; 0.43) | 0.19 | 0.21 (-0.05; 0.47) | 0.12 |
| Fat-free mass index, kg/m^2^ | 0.15 (-0.03; 0.33) | 0.10 | 0.04 (-0.15; 0.23) | 0.67 |
| Fat mass index, kg/m^2^ | 0.03 (-0.21; 0.15) | 0.76 | 0.03 (-0.22; 0.16) | 0.74 |
| Grip strength, kg | 0.50 (-0.65; 1.64) | 0.39 | 0.30 (-0.87; 1.47) | 0.62 |
| Chair Stand Test, count | 0.07 (-0.70; 0.84) | 0.86 | 0.71 (-0.09; 1.51) | 0.08 |
| Waist circumference, cm | 0.76 (-0.49; 2.00) | 0.24 | 0.98 (-0.28; 2.24) | 0.13 |
| Hip circumference, cm | 0.15 (-0.91; 1.22) | 0.78 | 0.57 (-0.50; 1.64) | 0.30 |
| Upper-arm circumference, cm | -0.08 (-0.57; 0.41) | 0.74 | 0.09 (-0.41; 0.59) | 0.73 |
| Protein intake, g | 17.70 (7.52; 27.89) | <0.01 | 16.24 (5.71; 26.76) | <0.01 |
| Energy intake, kcal | 315.61 (90.03; 541.19) | <0.01 | 388.39 (154.71; 622.07) | <0.01 |
| Physical activity, min/week | 23.92 (-94.95; 142.78) | 0.69 | -60.32 (-183.72; 63.08) | 0.34 |
| Higher EQ utility index and VAS indicate better quality of life. A positive mean difference reflects a beneficial effect for utility and VAS, while a negative mean difference reflects benefit for domain scores (scored 1–5). The model was adjusted for baseline imbalances (age, grip strength, protein intake, Charlson index), predefined covariates (sex, living alone, FEV1, smoking, physical activity), and the baseline outcome value. | | | | |
